# Expression of Concern on ‘Differential induction of Leishmania donovani bi-subunit topoisomerase I–DNA cleavage complex by selected flavones and camptothecin: activity of flavones against camptothecin-resistant topoisomerase I’

**DOI:** 10.1093/nar/gkag440

**Published:** 2026-04-29

This is an expression of concern about: Benu Brata Das, Nilkantha Sen, Amit Roy, Somdeb Bose Dasgupta, Agneyo Ganguly, Bikash Chandra Mohanta, Biswanath Dinda, Hemanta K. Majumder, Differential induction of Leishmania donovani bi-subunit topoisomerase I–DNA cleavage complex by selected flavones and camptothecin: activity of flavones against camptothecin-resistant topoisomerase I, Nucleic Acids Research, Volume 34, Issue 4, 1 February 2006, Pages 1121–1132, https://doi.org/10.1093/nar/gkj502

In October 2025, the Editors were notified of concerns regarding apparent duplications in two figures: lanes 4 and 5 in Figure 4A, and lanes 7–8 and 9–10 in Figure 7B.

The Editors have contacted the authors and are escalating to the institution. In the interim, we advise readers to examine the details of this study with particular care.

